# The validity of the Patient Health Questionnaire for screening depression in chronic care patients in primary health care in South Africa

**DOI:** 10.1186/s12888-015-0503-0

**Published:** 2015-05-23

**Authors:** Arvin Bhana, Sujit D Rathod, One Selohilwe, Tasneem Kathree, Inge Petersen

**Affiliations:** 1 School of Applied Human Sciences, University of KwaZulu-Natal, Durban, South Africa; 2 Department of Population Health, London School of Hygiene and Tropical Medicine, London, UK

**Keywords:** Questionnaire (PHQ-9), Validation studies, Depressive disorder, Chronic disease, South Africa, Mass screening, Diagnosis

## Abstract

**Background:**

People with chronic health conditions are known to have a higher prevalence of depressive disorder. The Patient Health Questionnaire (PHQ-9) is a widely-used screening tool for depression which has not yet been validated for use on chronic care patients in South Africa.

**Methods:**

A sample of 676 chronic care patients attending two primary health facilities in North West Province, South Africa were administered the PHQ-9 by field workers and a diagnostic interview (the Structured Clinical Interview for DSM-IV) (SCID) by clinical psychologists. The PHQ-9 and the PHQ-2 were evaluated against the SCID, as well as for sub-samples of patients who were being treated for HIV infection and for hypertension.

**Results:**

Using the SCID, 11.4 % of patients had major depressive disorder. The internal consistency estimate for the PHQ-9 was 0.76, with an area under the receiver operator curve (AUROC) of 0.85 (95 % CI 0.82–0.88), which was higher than the AURUC for the PHQ-2 (0.76, 95 % CI 0.73–0.79). Using a cut-point of 9, the PHQ-9 has sensitivity of 51 % and specificity of 94 %. The PHQ-9 AUROC for the sub-samples of patients with HIV and with hypertension were comparable (0.85 and 0.86, respectively).

**Conclusions:**

The PHQ-9 is useful as a screening tool for depression among patients receiving treatment for chronic care in a public health facility.

**Electronic supplementary material:**

The online version of this article (doi:10.1186/s12888-015-0503-0) contains supplementary material, which is available to authorized users.

## Background

An estimated 14 % of the global burden of disease can be attributed to neuropsychiatric disorders, primarily related to the disabling nature of depression and other common mental disorders [1, 2]. Depression has also been found to be co-morbid with a range of chronic diseases, including HIV/AIDS [3, 4] cardiovascular disorder (CVD) and diabetes [1, 5]. Large studies have shown that patients with chronic obstructive pulmonary disease, chronic renal failure and cerebrovascular disease compared with age-matched healthy controls were almost three times more likely to have depression, and twice as likely for patients with diabetes or hypertension [6, 7]. The WHO World Mental Health Survey in developed and developing countries [8] and the review of the evidence by the National Institute for Health and Clinical Excellence (NICE) guidance for depression in adults with a chronic physical health problem reports similar findings [9].

The co-morbidity of depression with chronic conditions is a public health concern due to their mutual reinforcement and synergistic clinical effects [7, 10, 11]. Evidence for the reciprocal relationship between depression and chronic physical health problems suggests a number of causal pathways, including emotional distress and poor sleep due to pain [12], the prospect of disability, [1] and changes in allostatic load whereby the ability of the body to adapt may be compromised due to ongoing tissue damage and degenerative changes [9]. Similarly, depression can also contribute to the development of physical health problems. Systematic reviews of 11 prospective cohort studies in health populations reveal that depression predicts later development of coronary heart disease [13] and stroke. Furthermore, major depressive disorder (MDD) can act as a precipitant for HIV infection as well be a consequence of HIV infection [14, 15]. One third of HIV-infected patients were found to be depressed in a recent screening study for depression in a high HIV burden primary health care clinic in South Africa study [16]. The burden of disease in South Africa is in the process of profound health transitions where communicable and non-communicable disorders coexist, and where chronic conditions such as cardiovascular disease, type 2 diabetes, cancer, chronic lung disease and depression are all predicted to increase substantially [17].

Treatment of depression is vital to improved adherence, social functioning and the disease course of chronic conditions [5, 14, 18, 19]. Early recognition and appropriate management of depression has the potential to improve adherence and impact on the social functioning and the disease progression of chronic conditions, thereby enhancing quality of life [5, 9, 18–20].

While identifying depressive disorder in primary care is recognized as important to effective treatment, only about half of patients with depressive disorder are detected by regular health care providers in high-income settings [21, 22]. In South Africa, this gap is far greater, with only one in four people with a common mental disorder receiving treatment [23]. With HIV transitioning to a chronic condition as a function of the roll-out of anti-retroviral treatment (ART) in South Africa, as well as the rising burden of NCDs (Non Communicable Diseases), which includes mental disorders [17], there is an imperative to integrate the treatment of depression with chronic disease management.

In the context of busy primary health care (PHC) clinics, the use of valid screening tools that facilitate assessment of depression by non-specialists would allow for greater depression detection and care, as well as being in line with the task-sharing model that has been embraced by the recent National Mental Health Policy Framework and Strategic Action Plan (2013–2020) in South Africa [24]. The Patients Health Questionnaire (PHQ-9) is a brief diagnostic and severity measure of depression, which has been widely used in research and clinical practice but has not been validated for use in South Africa on chronic care patients.

While there are numerous studies that report on the validity of the PHQ-9 in relation to various chronic illnesses, most of these studies fail to use an appropriate reference standard to evaluate the performance of the PHQ-9′s performance in identifying depression [25–28]. Only a few studies involving patients in chronic care have examined the PHQ-9 against a criterion standard. The PHQ-9 successfully screened post-stroke depression patients with operating characteristics similar or superior to other depression measures [29]. Similar findings are noted in the *Heart and Soul* study of depression among patients with coronary heart disease [30]. A meta-analysis that included chronic care conditions involving patients from cardiology, renal dialyses, brain injury and stroke facilities as well as general medical outpatients found the PHQ-9 acceptable as a diagnostic screening tool for major depression [31].

In sub-Saharan Africa, only a few studies have established the criterion validity of the PHQ-9 as a screening tool for depression [16, 26, 32, 33]. A meta-analysis establishing the range of optimal cut-off scores for diagnosing depression with the PHQ-9 notes that optimal cut-off scores range between 8 and 11 [34]. In low and middle income contexts, a positive screen for depression was defined as a score of ≥10 [32, 35].

This study aimed to validate the PHQ-9 as a screening tool for depression among chronic care patients attending two public primary health care facilities in South Africa. As a secondary objective, we validated the PHQ-2 - a subset of two questions drawn from the PHQ-9 – which is a simpler screening option that has been validated elsewhere [36]. The PHQ-2 has not been validated against a gold standard in sub-Saharan Africa, though it has been recommended as a valid and reliable tool for use in resource-constrained settings [28].

## Methods

### Setting and participants

The study was conducted in two primary health care clinics in the North West province of South Africa. The two clinics are pilot sites for a national Department of Health model for integrated chronic disease management, which adopts the collaborative chronic care model and services all chronic care patients at one service point [37].

### Study procedures

This validation study was nested within a larger facility detection survey designed to assess the detection and treatment of depression and alcohol use disorders by health care providers for adult attendees of primary health clinics. Patients who had come to the chronic care clinic were recruited from the consultation waiting room before their consultation with a clinician. In the waiting room, a field worker asked for volunteers to take part in a survey on depression and alcohol use disorders. The field worker directed interested individuals to a research assistant in a private consultation room. The research assistants who were recruited from the local communities had completed secondary school, were fluent in seTswana and English, and were trained in administering the PHQ-9 by a clinical psychologist. The research assistant assessed the patients for inclusion criteria, which were: age 18 years or older, clinic attendance for routine chronic disease services (*e.g.*, HIV, hypertension, diabetes) and ability to comprehend and complete study components in seTswana or English. Exclusion criteria included incapacity to provide informed consent (*e.g.*, less than 18 years of age, presence of severe intellectual disability or currently experiencing an acute medical issue, or in treatment for major depression). Eligible patients provided written informed consent to participate in the validation study. Patients with low levels of literacy could sign the informed consent form with an “X” after discussing the study with a study supervisor, and having the consent form read out to them.

Research assistants who administered the PHQ-9 were supervised by mid-level psychological counsellors with 4-year Bachelor’s degrees in psychology who were fluent in seTswana and trained by the seTswana-speaking clinical psychologist in the administration of the PHQ-9. These research assistants then orally administered the PHQ-9 screening tool, and entered the participant’s responses in a questionnaire application programmed onto a mobile handheld device [38]. In addition to the PHQ-9 screening instrument, the interview contained questions on socio-demographic characteristics, economic status, chronic care services received at the clinic, alcohol use and disability status as part of the larger study. Immediately after the conclusion of the screening interview, the research assistant directed the participant to another private consultation room for the diagnostic interview with a clinical psychologist. The research assistant did not appraise the participant or the psychologist of the participant’s PHQ-9 screening score. In each clinic, a clinical psychologist conducted the Structured Clinical Interview for DSM-IV (SCID) [39] diagnostic interview for a current episode of depressive disorder. One psychologist was fluent in both study languages, while the other was assisted by a seTswana-speaking mid-level trained psychological counsellor to conduct the diagnostic interviews. Most patients chose to have the interviews in seTswana with only a few who chose to be interviewed in English. At the conclusion of the diagnostic interview, any patient who 1) expressed suicidality (*i.e.* thoughts, plans, actions); or 2) was judged to have severe symptoms of depression by the clinical psychologist, was asked by the research assistant to provide consent for a referral to the consulting nurse in the clinic.

Ethical approval for the validation study was obtained from the University of KwaZulu-Natal Biomedical Research Ethics Committee (BE271/13). Ethical approval for the larger study was obtained from the University of KwaZulu-Natal Biomedical Research Ethics Committee (BE400/13), and the University of Cape Town, Faculty of Health Sciences, Human Research Ethics Committee (412/2011), and the World Health Organization Research Ethics Review Committee (RPC497).

### Assessments

The PHQ-9 asks patients to rate how often they were bothered by specific problems over the last two weeks. Each item is scored from 0 to 3 (0 = not at all; 1 = several days; 2 = more than half the days; and 3 = nearly every day) [36]. All nine items in the PHQ-9 are derived from *DSM-IV* criteria relevant for diagnosis of a current depressive episode [21]. The PHQ-2 is comprised of the first two questions of the PHQ-9, namely whether the patient has depressed mood and loss of interest (anhedonia). The PHQ-9 asks patients to rate how often they were bothered by specific problems over the last two weeks. Each item is scored from 0 to 3 according to the frequency of the problem. After a piloting process we adapted the response set to improve respondent understanding, such that “several days” was understood to be 1–7 days, “half the days” was understood to be 8–11 days and “nearly every day” was understood to be 12–14 days, as was the case in other validity studies in Africa [16, 26]. The PHQ-9 was translated into seTswana by a seTswana-speaking mental health professional and then back-translated by an independent seTswana-speaking clinical psychologist using the methodology described by Brislin [40] (Additional file 1). Due to time considerations and the logistics of administering the PHQ-9 twice to a clinic population, test-retest reliability was not done. Inter-rater reliability was also not completed for the same reasons.

The gold standard diagnostic interview was the depression module of the SCID-I to assess the participants for the presence or absence of major depressive disorder. The SCID is a semi-structured interview administered by a trained clinician who assesses a respondent for the presence or absence of a mental health disorder. At the time of the study, the more recent version of the SCID was not available. Nevertheless, the criteria for depression remains the same in the DSM-V as the DSM-IV version. According to the American Psychiatric Association “neither the core criterion symptoms applied to the diagnosis of major depressive episode nor the requisite duration of at least 2 weeks has changed from DSM-IV. Criterion A for a major depressive episode in DSM-V is identical to that of DSM-IV, as is the requirement for clinically significant distress or impairment in social, occupational, or other important areas of life” (p. 4) [41].

### Sample characteristics

Out of a total of 1321 eligible patients who participated in the larger facility survey, 1025 patients were attendees of the clinics where the validation study was conducted. Of these patients, a sub-sample of 676 patients participated in the validation study between February and April 2014. Participants who consented to the general study also consented to the PHQ9 validation study. The sub-sample size was limited by the availability of the clinical psychologists to conduct diagnostic interviews. Overall, there were 233 refusals to the larger study. Table 1 reflects the demographic characteristics of the 676 participants who completed both the SCID and PHQ-9 interviews. The participants were predominantly female (75.0 %), with mean age of 47.1 years (SD 13.1). Most participants (72.4 %) had completed 6 or more years of primary education. Participants could report multiple conditions for which they had been diagnosed and for which they were receiving services. The most common conditions reported were HIV infection (61.1 %), hypertension (51.0 %), diabetes (9.3 %), and tuberculosis (4.9 %). Of the 413 participants diagnosed with HIV and 345 patients diagnosed with hypertension, 118 participants were found to be co-morbid.

**Table 1:** Demographic and clinical characteristics chronic care patients (*n* = 676) in North West Province, South Africa

Description	*n* (%)
Sex	
Male	169 (25.0)
Female	507 (75.0)
Age (years)	
Median 47	IQR 37–56.5
	Range 18–88
Education	
None	52 (7.7)
Grades 1–5	135 (20.0)
Grades 6–12	478 (70.7)
>12 years	11 (1.6)
Chronic care service^a^	
HIV/AIDS	413 (61.1)
Hypertension	345 (51.0)
Diabetes	63 (9.3)
Tuberculosis	33 (4.9)
Asthma	24 (3.6)
Epilepsy	23 (3.4)
Arthritis	23 (3.4)
Other Mental Health^b^	17 (2.5)
Heart problems	6 (0.9)
Chronic obstructed pulmonary disorder	2 (0.30)
Number of chronic care services	
1	399 (59.0)
2	237 (35.1)
3	38 (5.3)
> = 4	2 (0.3)

*IQR* interquartile range

^a^Participants can report >1 chronic service, total is >100 %

^b^For example, Schizophrenia and Bipolar Mood Disorders

### Data analyses

First, we described the socio-demographic and clinical characteristics of participants recruited for this validation study and proportion of patients receiving a depression diagnosis on the SCID. We evaluated the internal consistency of the PHQ-9 by calculating the Cronbach alpha. Next, we used the screening scores from the PHQ-9 interview to construct a receiver operating characteristic (ROC) curve and calculated the area under the ROC (AUROC) using the SCID as the gold standard. The AUROC provides a summary measure of a screening tool’s sensitivity and specificity, relative to the gold standard diagnostic, across the entire range of screening scores. An AUROC score of 0.5 is consistent with a screening tool that is no better than chance, and a score of 1.0 indicates a perfectly accurate screening tool. We also calculated the AUROC for the PHQ2 and compared it to the AUROC for the PHQ-9. To explore potential heterogeneity of the validity of the PHQ-9, we also repeated the AUROC analysis with two (overlapping) subsets of participants who reported that their clinic attendance was for ongoing care with HIV infection and with hypertension. For all AUROC calculations, we report exact binomial 95 % confidence intervals (95 % CI).

We completed all analyses using Stata 13.1 (StataCorp, College Station, USA); we used the ‘roctab’ command for analysis of the individual AUCs, and ‘roccomp’ for the comparison of the PHQ-9 and the PHQ-2.

## Results

### Descriptive results

In the SCID diagnostic interview, more than one in ten (11.47 %) participants were diagnosed as currently experiencing a major depressive episode. The mean PHQ-9 score for those who were SCID-positive (PHQ-9 score 9.4, SD 5.3) was substantially higher than for those who were SCID-negative (PHQ-9 score 3.2, SD 3.1). For those with an HIV diagnosis, 12.6 % were SCID-positive, while 9.9 % of those with hypertension were also SCID-positive. No participant experienced any adverse events during either interview. The study psychologist referred 69 participants to the clinic on-call nurse, following disclosure of suicidal ideation, planning or attempts (Table 2).

**Table 2:** Performance of the PHQ-9 and PHQ-2 to detect major depressive disorder among chronic care patients (*n* = 676)

	PHQ-9					PHQ-2		
Cut point	Sensitivity (%)	Specificity (%)	Correctly classified (%)	LR + ^a^	LR-^b^	Sensitivity (%)	Specificity (%)	Correctly classified (%)
(≥0)	100.0	0.0	11.4	1.000		100.0	0.0	11.4
(≥1)	100.0	21.4	30.3	1.2718	0.0000	80.5	57.9	60.5
(≥2)	97.4	37.1	43.9	1.5476	0.0701	59.7	83.5	80.8
(≥3)	90.9	52.4	56.8	1.9107	0.1734	39.0	91.7	85.7
(≥4)	87.0	63.3	66.0	2.3691	0.2053	19.5	97.8	88.9
(≥5)	80.5	71.8	72.8	2.8539	0.2714	9.1	99.3	89.1
(≥6)	71.4	79.1	78.2	3.4229	0.3611	0.0	100.0	88.6
(≥7)	64.9	85.6	83.3	4.5228	0.4094			
(≥8)	53.2	90.6	86.4	5.6955	0.5157			
(≥9)	49.3	93.7	88.6	7.7792	0.5408			
(≥10)	41.6	96.5	90.2	11.8540	0.6056			
(≥11)	36.4	98.2	91.1	19.8016	0.6483			
(≥12)	32.5	98.5	91.0	21.6090	0.6856			
(≥13)	26.0	98.5	90.2	17.2872	0.7516			
(≥14)	24.7	98.8	90.4	21.1150	0.7622			
(≥15)	19.5	99.5	90.4	38.8963	0.8092			
(≥16)	14.3	99.7	89.9	42.7863	0.8600			
(≥17)	9.1	99.7	89.4	27.2276	0.9121			
(≥19)	9.1	99.4	89.5	54.4541	0.9106			
(≥20)	3.9	99.8	88.9	23.3375	0.9626			
(≥21)	2.6	99.8	88.8	15.5583	0.9757			
(≥22)	1.3	100.0	88.8		0.9870			
(≥22)	0.0	100.0	88.6		1.0000			

The SCID depression module was used as the diagnostic reference standard

^a^Likelihood Ratio Positive

^b^Likelihood Ratio Negative

Internal consistency of the PHQ-9 revealed a Cronbach alpha of 0.76.

### Sensitivity and specificity of the PHQ-9 and PHQ-2

With the full study sample, the PHQ-9 showed reasonably high validity (AUROC 0.85, 95 % CI 0.82–0.88). With a cut point of ≥9, the PHQ-9 had sensitivity of 49 % and specificity of 94 %. The likelihood ratio of a person testing positive for depression was seven times more likely at this cut point (7.7792). The Overall Correct Classification (OCC) rate of 0.886 was also the best at a cutoff of 9 and above. With this cut point, the positive and negative predictive values were 50 % and 94 %, respectively. Youden’s Index (Youden’s J) identifies a cut point of ≥5, with a maximum value of sensitivity- (1-specificity) of 52.3 %.

The validity of the PHQ-9 was similar for the subsamples of 413 patients receiving services for HIV infection (AUROC 0.85, 95 % CI 0.81–0.88) and the 345 patients receiving services for hypertension (AUROC 0.85, 95 % CI 0.82–0.90).

**Fig. 1:**
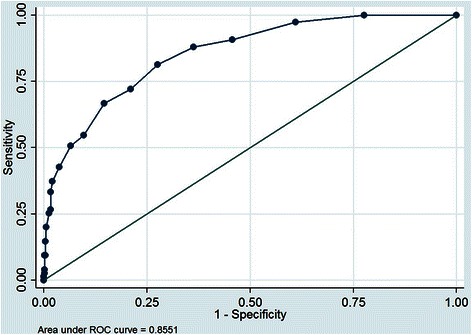
Receiver operating characteristic (ROC) curve for PHQ-9

**Fig. 2:**
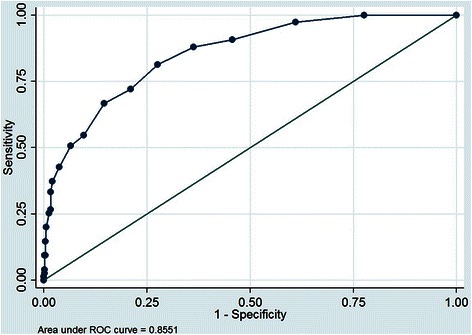
Receiver operating characteristic (ROC) curve for PHQ-2

**Fig. 3:**
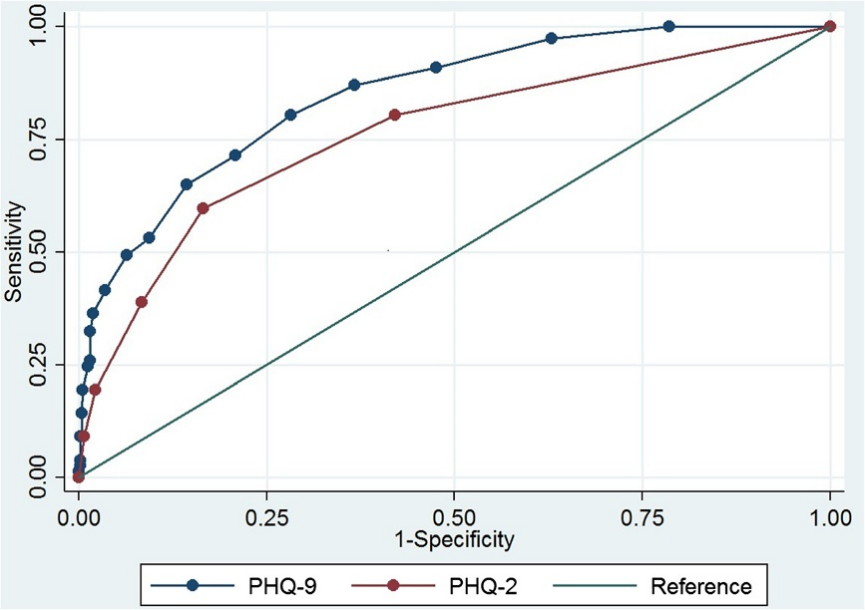
Receiver operating characteristic (ROC) curve comparing PHQ-9 to PHQ-2

While the PHQ-2 appeared to be a valid screening tool (AUROC 0.76, 95 % CI 0.73–0.79), its AUROC was significantly lower than for the PHQ-9 (*p* < 0.0001). With a cut point of ≥2, the PHQ-2 had sensitivity of 60 % and specificity of 84 %, correctly classifying 81 % of the population at this level (Figs. 1, 2 and 3, Tables 3, 4 and 5).

**Table 3:** Item-level discrimination PHQ-9 (*N* = 676)

Item	Item-test correlation	Item-rest correlation	Average Inter-item covariance	Item Alpha
Phq1	0.578	0.429	0.144	0.734
Phq2	0.649	0.491	0.133	0.724
Phq3	0.619	0.440	0.135	0.734
Phq4	0.655	0.509	0.133	0.721
Phq5	0.620	0.451	0.135	0.732
Phq6	0.602	0.465	0.142	0.723
Phq7	0.587	0.466	0.147	0.730
Phq8	0.347	0.250	0.170	0.757
Phq9	0.546	0.420	0.150	0.736
Overall			0.143	0.756

**Table 4:** Performance of the PHQ-9 in Detecting Depression in HIV positive patients (*n* = 413) in North West Province, South Africa

Cut-point	Sensitivity %	Specificity %	Percent correctly classified	LR+	LR-
(≥0)	100.00	0.00	12.59	1.000	
(≥1)	100.00	21.05	30.99	1.2667	0.0000
(≥2)	98.08	33.80	41.89	1.4814	0.0569
(≥3)	88.46	49.31	54.24	1.7451	0.2340
(≥4)	84.26	61.22	64.16	2.1819	0.2513
(≥5)	82.69	68.42	70.22	2.6186	0.2530
(≥6)	76.92	77.29	77.24	3.3865	0.2986
(≥7)	71.15	83.93	82.32	4.4287	0.3437
(≥8)	57.69	88.92	84.99	5.2067	0.4758
(≥9)	57.69	92.80	88.38	8.0104	0.4559
(≥10)	48.08	95.57	89.59	10.4874	0.5433
(≥11)	40.38	97.51	90.31	16.1987	0.6114
(≥12)	36.54	98.06	90.31	18.8434	0.6472
(≥13)	28.85	98.06	89.35	14.8764	0.7256
(≥14)	26.92	98.61	89.59	19.4385	0.7410
(≥15)	23.08	99.45	89.83	41.6359	0.7735
(≥16)	17.31	99.72	89.35	62.4808	0.8292
(≥19)	11.54	99.72	88.62	41.6539	0.8871
(≥20)	5.77	99.72	87.89	20.8269	0.9449
(≥21)	3.85	99.72	87.65	13.8846	0.9642
(≥22)	1.92	100	87.65		0.9808
(≥22)	0.00	100	87.41		1.0000
Observations	ROC Area under the curve	Std. Error	95 % Confidence Interval		
413	0.8488	0.0293	0.81	0.88

**Table 5:** Performance of the PHQ-9 in Detecting Depression in Patients with hypertension (*n* = 345) in North West Province, South Africa

Cut-point	Sensitivity %	Specificity %	Percent correctly classified	LR+	LR-
(≥0)	100.00	0.00	9.86	1.000	
(≥1)	100.00	20.58	28.41	1.2591	0.0000
(≥2)	97.06	37.03	43.19	1.5408	0.0789
(≥3)	97.06	51.77	56.23	2.0124	0.0568
(≥4)	91.18	62.38	65.22	2.4236	0.1414
(≥5)	79.41	72.35	73.04	2.8718	0.2846
(≥6)	67.65	80.39	79.13	3.4489	0.4025
(≥7)	64.71	85.85	83.77	4.5735	0.4111
(≥8)	52.94	91.96	88.12	6.5859	0.5117
(≥9)	44.12	94.53	89.57	8.0709	0.5911
(≥10)	38.42	98.07	92.17	19.8186	0.6298
(≥11)	32.35	99.36	92.75	50.3086	0.6808
(≥12)	29.41	99.36	92.46	45.7351	0.7105
(≥14)	23.53	99.36	91.88	36.5881	0.7697
(≥15)	17.65	99.68	91.59	51.8828	0.8262
(≥16)	11.76	99.68	91.01	36.5885	0.8852
(≥17)	8.82	99.68	90.72	27.4414	0.9147
(≥19)	8.82	100	91.01		0.9118
(≥20)	2.94	100	90.43		0.9706
(≥20)	0.00	100	90.14		0.9706
Observations	ROC Area under the curve	Std. Error	95 % Confidence Interval		
345	0.8596	0.0309	0.82	0.90	

## Discussion

The health care burden associated with the burgeoning chronic care population in South Africa makes this a timely and important focus as individuals with depression are less likely to be treatment adherent or engage in health enhancing behavior change to promote healthy lifestyles [5, 18–20]. Given that identifying depression in chronic care patients can be a diagnostic challenge in busy clinics, a short and valid screening tool can assist in the identification of patients with depression.

A meta-analysis establishing the range of optimal cut-off scores for diagnosing depression with the PHQ-9 notes that optimal cut-off scores range between 8 and 11 [34]. In this study, a cut-point of ≥9 yielded a fairly low sensitivity (49 %) in comparison to sensitivity indices of 78.7 % and 89.6 %, respectively in other similar validity studies in sub-Saharan Africa [16, 32], However, higher specificity (94 %) was noted in the present study relative to the only other South African study (83.4 %) [16] and was the same as that found for HIV patients in Cameroon [26]. A prospective study of psychosocial factors and health outcomes in patients with a diagnosis of coronary heart disease also showed similar sensitivity and specificity to the present study [30].

Similar to the PHQ-9, the PHQ-2 had lower sensitivity (60 %) than specificity (84 %) and may be explained in the same way as that for the PHQ-9 given that these data are drawn from the same sample. The PHQ-2 remains a valid option for use, particularly in time-constrained settings. The trade-off between sensitivity and specificity using the PHQ-2 is more substantial than for the PHQ-9. Selecting a high cut-off score when using the PHQ-2 would enable clinicians to screen a large number of patients, but then to refer a relatively modest number of patients – who are likely true cases - for confirmation of a diagnosis of depression.

The strength of this study is that it is one of the few studies to consider the validity of the PHQ-9 in sub-Saharan Africa, and the first to validate the PHQ-9 or the PHQ-2 for a chronic care population in South Africa. Limitations of this study include that the participants were drawn from only two clinics in North West province, and were predominantly female. Therefore, it may not be possible to generalize our results to other populations. We were unable to establish test-retest reliability due to time considerations and the burden it would impose on the public health clinics. On the same basis, we were unable to establish inter-rater reliability of administration of the gold standard instrument. For logistic reasons we were also unable to randomize the order of interviews as participants with depression would be more likely to be detected in the diagnostic interview than in the screening interview. Further, a willingness to disclose feelings of distress in the diagnostic interview may have been heightened after completing the screening interview. This ‘order’ effect may have biased our AUROC results to be lower than the true values.

## Conclusions

The brevity of the scale, ease of administration and its concurrent validity with the SCID suggests that the PHQ-9 can be a valuable instrument for identifying co-morbid depression in patients with chronic conditions using a cut-point of ≥9. It is possible that the abbreviated PHQ-2 could be used in a busy primary care clinic, but would need to be followed up with a full assessment. Identification is the first step towards closing the treatment gap for depression and advancing the agenda of integrated chronic disease management in South African public health facilities. Further research is needed in understanding how patients with low levels of understanding of depression as a legitimate disorder and recognition of depressive symptoms are likely to respond to screening tools such as the PHQ-9.

## Additional file

Additional file 1:
**PHQ-9 and treatment{ TC “13 items; (c) Pfizer 1999; History question adapted from MINI” \l 5 n }.**

## References

[1] Prince M, Patel V, Saxena S, Maj M, Maselko J, Phillips MR, Rahman A (2007). No health without mental health. Lancet.

[2] Murray CJ, Vos T, Lozano R, Naghavi M, Flaxman AD, Michaud C, Ezzati M, Shibuya K, Salomon JA, Abdalla S (2012). Disability-adjusted life years (DALYs) for 291 diseases and injuries in 21 regions, 1990–2010: a systematic analysis for the Global Burden of Disease Study 2010. Lancet.

[3] Brandt R (2009). The mental health of people living with HIV/AIDS in Africa: a systematic review. Afr J AIDS Res.

[4] Owe-Larsson B, Sall L, Salamon E, Allgulander C (2009). HIV infection and psychiatric illness. Afr J Psychiatry.

[5] Ziegelstein RC, Fauerbach JA, Stevens SS, Romanelli J, Richter DP, Bush DE (2000). Patients with depression are less likely to follow recommendations to reduce cardiac risk during recovery from a myocardial infarction. Arch Intern Med.

[6] Egede LE (2007). Major depression in individuals with chronic medical disorders: prevalence, correlates and association with health resource utilization, lost productivity and functional disability. Gen Hosp Psychiatry.

[7] Moussavi S, Chatterji S, Verdes E, Tandon A, Patel V, Ustun B (2007). Depression, chronic diseases, and decrements in health: results from the World Health Surveys. Lancet.

[8] von Korff M, Scott K, Gureje O (2009). Global Perspectives on Mental–Physical Comorbidity in the WHO Mental Health Surveys.

[9] National Collaborating Centre for Mental H (2010). National Institute for Health and Clinical Excellence: Guidance. Depression in Adults with a Chronic Physical Health Problem: Treatment and Management. edn.

[10] Thombs BD, Ziegelstein RC, Whooley MA (2008). Optimizing detection of major depression among patients with coronary artery disease using the patient health questionnaire: data from the heart and soul study. J Gen Intern Med.

[11] Breuer E, Myer L, Struthers H, Joska J (2011). HV/AIDS and mental health research in sub-Saharan Africa: a systematic review. Afr J AIDS Res.

[12] Dworkin SF, Von Korff M, LeResche L (1990). Multiple pains and psychiatric disturbance. An epidemiologic investigation. Arch Gen Psychiatry.

[13] Nicholson A, Kuper H, Hemingway H (2006). Depression as an aetiologic and prognostic factor in coronary heart disease: a meta-analysis of 6362 events among 146 538 participants in 54 observational studies. Eur Heart J.

[14] Simoni JM, Safren SA, Manhart LE, Lyda K, Grossman CI, Rao D, Mimiaga MJ, Wong FY, Catz SL, Blank MB (2011). Challenges in addressing depression in HIV research: assessment, cultural context, and methods. AIDS Behav.

[15] Smit J, Myer L, Middelkoop K, Seedat S, Wood R, Bekker LG, Stein DJ (2006). Mental health and sexual risk behaviours in a South African township: a community-based cross-sectional study. Public Health.

[16] Cholera R, Gaynes BN, Pence BW, Bassett J, Qangule N, Macphail C, Bernhardt S, Pettifor A, Miller WC (2014). Validity of the patient health questionnaire-9 to screen for depression in a high-HIV burden primary healthcare clinic in Johannesburg, South Africa. J Affect Disord.

[17] Mayosi BM, Flisher AJ, Lalloo UG, Sitas F, Tollman S, Bradshaw D (2009). The burden of non-communicable diseases in South Africa. Lancet.

[18] Gonzalez JS, Batchelder AW, Psaros C, Safren SA (2011). Depression and HIV/AIDS treatment nonadherence: a review and meta-analysis. J Acquir Immune Defic Syndr.

[19] Starace F, Ammassari A, Trotta MP, Murri R, De Longis P, Izzo C, Scalzini A, d’Arminio Monforte A, Wu AW, Antinori A (2002). Depression is a risk factor for suboptimal adherence to highly active antiretroviral therapy. J Acquir Immune Defic Syndr.

[20] Leserman J (2008). Role of depression, stress, and trauma in HIV disease progression. Psychosom Med.

[21] Wittkampf K, van Ravesteijn H, Baas K, van de Hoogen H, Schene A, Bindels P, Lucassen P, van de Lisdonk E, van Weert H (2009). The accuracy of Patient Health Questionnaire-9 in detecting depression and measuring depression severity in high-risk groups in primary care. Gen Hosp Psychiatry.

[22] Gilbody S, Richards D, Barkham M (2007). Diagnosing depression in primary care using self-completed instruments: UK validation of PHQ-9 and CORE-OM. Br J Gen Pract.

[23] Seedat S, Williams DR, Herman AA, Moomal H, Williams SL, Jackson PB, Myer L, Stein DJ (2009). Mental health service use among South Africans for mood, anxiety and substance use disorders. S Afr Med J.

[24] Department of Health (2013). National Mental Health Policy Framework and Strategic Plan 2013–2020.

[25] Wu SF (2014). Rapid screening of psychological well-being of patients with chronic illness: reliability and validity test on WHO-5 and PHQ-9 scales. Depress Res Treat..

[26] Pence BW, Gaynes BN, Atashili J, O’Donnell JK, Tayong G, Kats D (2012). Validity of an interviewer-administered patient health questionnaire-9 to screen for depression in HIV-infected patients in Cameroon. J Affect Disord.

[27] Monahan PO, Shacham E, Reece M, Kroenke K, Ong’or WO, Omollo O (2009). Validity/reliability of PHQ-9 and PHQ-2 depression scales among adults living with HIV/AIDS in western Kenya. J Gen Intern Med.

[28] Omoro SA, Fann JR, Weymuller EA, Macharia IM, Yueh B (2006). Swahili translation and validation of the Patient Health Questionnaire-9 depression scale in the Kenyan head and neck cancer patient population. Int J Psychiatry Med.

[29] Williams LS, Brizendine EJ, Plue L, Bakas T, Tu W, Hendrie H (2005). Performance of the PHQ-9 as a screening tool for depression after stroke. Stroke.

[30] McManus D, Pipkin SS, Whooley MA (2005). Screening for depression in patients with coronary heart disease (data from the Heart and Soul Study). Am J Cardiol.

[31] Gilbody S, Richards D, Brealey S, Hewitt C (2007). Screening for depression in medical settings with the patient health questionnaire (PHQ): a diagnostic meta-analysis. J Gen Intern Med.

[32] Adewuya AO, Ola BA, Afolabi OO (2006). Validity of the patient health questionnaire (PHQ-9) as a screening tool for depression amongst Nigerian university students. J Affect Disord.

[33] Weobong B, Akpalu B, Doku V, Owusu-Agyei S, Hurt L, Kirkwood B (2009). The comparative validity of screening scales for postnatal common mental disorder in Kintampo. Ghana. J Affect Disord..

[34] Manea L, Gilbody S, McMillan D (2012). Optimal cut-off score for diagnosing depression with the Patient Health Questionnaire (PHQ-9): a meta-analysis. CMAJ.

[35] Akena D, Joska J, Obuku EA, Amos T, Musisi S, Stein DJ (2012). Comparing the accuracy of brief versus long depression screening instruments which have been validated in low and middle income countries: a systematic review. BMC Psychiatry..

[36] Kroenke K, Spitzer RL, Williams JB, Lowe B (2010). The patient health questionnaire somatic, anxiety, and depressive symptom scales: a systematic review. Gen Hosp Psychiatry.

[37] Wagner EH, Austin BT, Von Korff M (1996). Organizing care for patients with chronic illness. Milbank Q.

[38] Mobenzi. [http://mobenzi.com/]

[39] First MB, Spitzer RL, Williams JBW, Gibbon M (1995). Structured Clinical Interview for DSM-IV (SCID).

[40] Brislin RW (1970). Back-translation for cross-cultural research. J Cross-Cult Psychol.

[41] Highlights of changes from DSM-IV-TR to DSM-5. [http://www.dsm5.org/Documents/changes%20from%20dsm-iv-tr%20to%20dsm-5.pdf]

