# Supplementary material for: The validity of the Patient Health Questionnaire for screening depression in chronic care patients in primary health care in South Africa
Source: BMC Psychiatry. 2015 May 23;15:118. doi: 10.1186/s12888-015-0503-0 (PMC4446842; doi:10.1186/s12888-015-0503-0)
Supplement: Additional file 1: — PHQ-9 and treatment{ TC “13 items; (c) Pfizer 1999; History question adapted from MINI” \l 5 n }. [file 12888_2015_503_MOESM1_ESM.docx]

**Additional file 1**

| PHQ-9 and treatment | | | | |
| --- | --- | --- | --- | --- |
| Ke tla go botsa dipotso di se kae mme ke kopa o di arabe o nagane kana o gopole ka dilo tse di etsagetseng mo dibekeng tse pedi tse di fetileng. Go etsagagetse ga kae gore o ikutlwe o tshwenngwa ke mangwe a mathata a latelang? | | | | |
|  | O iphitlhela o nale kgatlhego e ko tlase go etsa dintho **mo dibekeng tse pedi tse di fetileng**. | Ga go ise go nketsagalele | 0 | **PHQ1** |
|  |  | Kwa tlase ga malatsi a le bosupa (7) | 1 |  |
|  |  | Go feta malatsi a le supa (7) | 2 |  |
|  |  | Go batlile e ka nna letsatsi le letsatsi | 3 |  |
|  | O utlwa mowa oo le ko tlase kana maikutlo a gago a le kwa tlase, o latlhegetswe ke tsholofelo **mo dibekeng tse pedi tse di fetileng** | Ga go ise go nketsagalele | 0 | **PHQ2** |
|  |  | Kwa tlase ga malatsi a le bosupa (7) | 1 |  |
|  |  | Go feta malatsi a le supa (7) | 2 |  |
|  |  | Go batlile e ka nna letsatsi le letsatsi | 3 |  |
|  | O sokola go ka robala/thulamela kana go tswelela o ntse o robetse/thulametse, kana o robala mo go feteletseng **mo dibekeng tse pedi tse di fetileng** | Ga go ise go nketsagalele | 0 | **PHQ3** |
|  |  | Kwa tlase ga malatsi a le bosupa (7) | 1 |  |
|  |  | Go feta malatsi a le supa (7) | 2 |  |
|  |  | Go batlile e ka nna letsatsi le letsatsi | 3 |  |
|  | O utlwa o kgathetse kana o sena thata/nonofo/maatla **mo dibekeng tse pedi tse di fetileng** | Ga go ise go nketsagalele | 0 | **PHQ4** |
|  |  | Kwa tlase ga malatsi a le bosupa (7) | 1 |  |
|  |  | Go feta malatsi a le supa (7) | 2 |  |
|  |  | Go batlile e ka nna letsatsi le letsatsi | 3 |  |
|  | O sena kgatlhego ya go ja kana o ja mo go feteletseng **mo dibekeng tse pedi tse di fetileng** | Ga go ise go nketsagalele | 0 | **PHQ5** |
|  |  | Kwa tlase ga malatsi a le bosupa (7) | 1 |  |
|  |  | Go feta malatsi a le supa (7) | 2 |  |
|  |  | Go batlile e ka nna letsatsi le letsatsi | 3 |  |
|  | **Mo dibekeng tse pedi tse di fetileng**, o utlwa o sa kgotsofalele se o leng sone- kana o icheba/ipona jaaka motho yo o tlhotsweng kana o bona wena kana o swabisitse ba kwa ga lona. | Ga go ise go nketsagalele | 0 | **PHQ6** |
|  |  | Kwa tlase ga malatsi a le bosupa (7) | 1 |  |
|  |  | Go feta malatsi a le supa (7) | 2 |  |
|  |  | Go batlile e ka nna letsatsi le letsatsi | 3 |  |
|  | O sokola go beya kelelo ya gago mo selong se le sengwe jaaka go bala pampiri ya ditaba/koranta (newspapwer) kana go shebelela TV (television) **mo dibekeng tse pedi tse di fetileng** | Ga go ise go nketsagalele | 0 | **PHQ7** |
|  |  | Kwa tlase ga malatsi a le bosupa (7) | 1 |  |
|  |  | Go feta malatsi a le supa (7) | 2 |  |
|  |  | Go batlile e ka nna letsatsi le letsatsi | 3 |  |
|  | O tsamaya kana o bua ka bonya mo go ka tswang go lemogilwe/bonwe ke batho ba bangwe. Kana se se farologaneng- o dula o sa iketla mo o fitlhelang e bile o tsamaya tsamaya fela, go nale jaaka pele **mo dibekeng tse pedi tse di fetileng** | Ga go ise go nketsagalele | 0 | **PHQ8** |
|  |  | Kwa tlase ga malatsi a le bosupa (7) | 1 |  |
|  |  | Go feta malatsi a le supa (7) | 2 |  |
|  |  | Go batlile e ka nna letsatsi le letsatsi | 3 |  |
|  | O nagana gore go ka bo go le botoka (betere) fa o ne o sule/tlhokafetse kana go ka ikgobatsa ka tsela nngwe fela **mo bekeng tse pedi tse di fetileng** | Ga go ise go nketsagalele | 0 | **PHQ9** |
|  |  | Kwa tlase ga malatsi a le bosupa (7) | 1 |  |
|  |  | Go feta malatsi a le supa (7) | 2 |  |
|  |  | Go batlile e ka nna letsatsi le letsatsi | 3 |  |
|  | Total for PHQ1-PHQ9 | 🖎________ |  | **PHQTOT** |
